# Bis(dimethyl­ammonium) 2,5-dihy­droxy­benzene-1,4-disulfonate

**DOI:** 10.1107/S1600536812003406

**Published:** 2012-01-31

**Authors:** Shan Gao, Seik Weng Ng

**Affiliations:** a Key Laboratory of Functional Inorganic Material Chemistry, Ministry of Education, Heilongjiang University, Harbin 150080, People’s Republic of China; b Department of Chemistry, University of Malaya, 50603 Kuala Lumpur, Malaysia; c Chemistry Department, Faculty of Science, King Abdulaziz University, PO Box 80203 Jeddah, Saudi Arabia

## Abstract

In the crystal of the title salt, 2C_2_H_8_N^+^·C_6_H_4_O_8_S_2_^2−^, the anion lies on a center of inversion. The dimethyl­ammonium cation forms one N—H⋯O hydrogen bond and another bifurcated N—H⋯O hydrogen bond. The hy­droxy group links with the sulfonyl group *via* an inter­molecular O—H⋯O hydrogen bond. These N—H⋯O and O—H⋯O hydrogen bonds generate a three-dimensional network.

## Related literature

For the diethyl­ammonium salt, see: Solans *et al.* (1982 ▶).
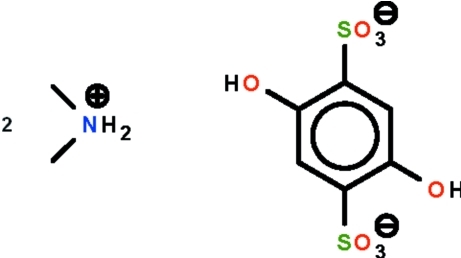


## Experimental

### 

#### Crystal data

2C_2_H_8_N^+^·C_6_H_4_O_8_S_2_^2−^
*M* *_r_* = 360.40
Monoclinic, 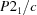

*a* = 8.0136 (12) Å
*b* = 12.2741 (19) Å
*c* = 9.2061 (16) Å
β = 115.268 (5)°
*V* = 818.9 (2) Å^3^
*Z* = 2
Mo *K*α radiation
μ = 0.36 mm^−1^
*T* = 293 K
0.25 × 0.20 × 0.15 mm

#### Data collection

Rigaku R-AXIS RAPID IP diffractometer
Absorption correction: multi-scan (*ABSCOR*; Higashi, 1995 ▶) *T*_min_ = 0.770, *T*_max_ = 1.000
7785 measured reflections
1849 independent reflections
1675 reflections with *I* > 2σ(*I*)
*R*_int_ = 0.037

#### Refinement

*R*[*F* ^2^ > 2σ(*F* ^2^)] = 0.042
*wR*(*F* ^2^) = 0.115
*S* = 1.07
1849 reflections
112 parameters
3 restraints
H atoms treated by a mixture of independent and constrained refinement
Δρ_max_ = 0.78 e Å^−3^
Δρ_min_ = −0.25 e Å^−3^

### 

Data collection: *RAPID-AUTO* (Rigaku, 1998 ▶); cell refinement: *RAPID-AUTO*; data reduction: *CrystalClear* (Rigaku/MSC, 2002 ▶); program(s) used to solve structure: *SHELXS97* (Sheldrick, 2008 ▶); program(s) used to refine structure: *SHELXL97* (Sheldrick, 2008 ▶); molecular graphics: *X-SEED* (Barbour, 2001 ▶); software used to prepare material for publication: *publCIF* (Westrip, 2010 ▶).

## Supplementary Material

Crystal structure: contains datablock(s) global, I. DOI: 10.1107/S1600536812003406/xu5456sup1.cif

Structure factors: contains datablock(s) I. DOI: 10.1107/S1600536812003406/xu5456Isup2.hkl

Supplementary material file. DOI: 10.1107/S1600536812003406/xu5456Isup3.cml

Additional supplementary materials:  crystallographic information; 3D view; checkCIF report

## Figures and Tables

**Table 1:** Hydrogen-bond geometry (Å, °)

*D*—H⋯*A*	*D*—H	H⋯*A*	*D*⋯*A*	*D*—H⋯*A*
O4—H4⋯O3^i^	0.83 (1)	1.85 (1)	2.670 (2)	175 (2)
N1—H1⋯O1	0.88 (1)	2.13 (2)	2.866 (2)	140 (2)
N1—H1⋯O1^ii^	0.88 (1)	2.21 (2)	2.921 (2)	138 (2)
N1—H2⋯O2^iii^	0.89 (1)	2.07 (2)	2.837 (2)	143 (3)

Symmetry codes: (i) 
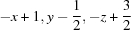
; (ii) 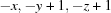; (iii) 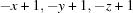.

**Fig. 1.**
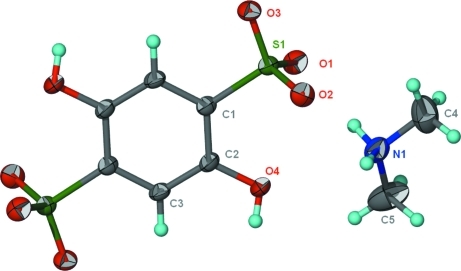
Thermal ellipsoid plot (Barbour, 2001) of 2(CH3)2NH2.C6H2(OH)2(SO3)2 at the 50% probability level; hydrogen atoms are drawn as spheres of arbitrary radius.

## supplementary crystallographic information

### Comment

Bis(diethylammonium) 2,5-dihydroxy-1,4-benzenedisulfonate is a commercial pharmacological chemical whose crystal structure has been described (Solans *et al.*, 1982). The title dimethyammonium salt (Scheme I) is an unexpected product of a hydrothermal synthesis involving 2,5-dihydroxy-1,4-benzenesulfonate in DMS solvent; the dimethylammonium cation probably resulted from the decomposition of DMF. The anion lies on a center-of-inversion (Fig. 1). The dimethylammonium cation forms one N–H···O hydrogen bond and another bifurcated hydrogen bond. These N–H···O and O–H···O hydrogen bonds generate a three-dimensional network (Table 1).

### Experimental

DMF (8 ml), magnesium hydroxide (1 mmol) and 1,4-dihydroxy-2,5-benzenedisulfonic acid (1 mmol) were heated in a 23-ml, Teflon-lined, stainless-stell Parr bomb at 413 K for 3 days. Colorless crystals were isolated from the cool vessel.

### Refinement

The carbon-bound H-atoms were placed in a calculated position (C–H 0.93 and 0.96 Å) and were included in the refinement in the riding model approximation, *U*(H) set to 1.2*U*(C). The amino and hydroxy H-atoms were located in a difference Fourier map, and were refined with distance restraints of N–H 0.88±0.01 Å, O–H 0.84±0.01 Å; their temperature factors were refined.

### Crystal data

**Table:**

2C_2_H_8_N^+^·C_6_H_4_O_8_S_2_^2^^−^	*F*(000) = 380
*M**_r_* = 360.40	*D*_x_ = 1.462 Mg m^−^^3^
Monoclinic, *P*2_1_/*c*	Mo *K*α radiation, λ = 0.71073 Å
Hall symbol: -P 2ybc	Cell parameters from 5427 reflections
*a* = 8.0136 (12) Å	θ = 3.3–27.4°
*b* = 12.2741 (19) Å	µ = 0.36 mm^−^^1^
*c* = 9.2061 (16) Å	*T* = 293 K
β = 115.268 (5)°	Prism, colorless
*V* = 818.9 (2) Å^3^	0.25 × 0.20 × 0.15 mm
*Z* = 2	

### Data collection

**Table:**

Rigaku R-AXIS RAPID IP diffractometer	1849 independent reflections
Radiation source: fine-focus sealed tube	1675 reflections with *I* > 2σ(*I*)
graphite	*R*_int_ = 0.037
ω scan	θ_max_ = 27.4°, θ_min_ = 3.3°
Absorption correction: multi-scan (*ABSCOR*; Higashi, 1995)	*h* = −9→10
*T*_min_ = 0.770, *T*_max_ = 1.000	*k* = −15→15
7785 measured reflections	*l* = −11→11

### Refinement

**Table:**

Refinement on *F*^2^	Primary atom site location: structure-invariant direct methods
Least-squares matrix: full	Secondary atom site location: difference Fourier map
*R*[*F*^2^ > 2σ(*F*^2^)] = 0.042	Hydrogen site location: inferred from neighbouring sites
*wR*(*F*^2^) = 0.115	H atoms treated by a mixture of independent and constrained refinement
*S* = 1.07	*w* = 1/[σ^2^(*F*_o_^2^) + (0.0776*P*)^2^ + 0.1341*P*] where *P* = (*F*_o_^2^ + 2*F*_c_^2^)/3
1849 reflections	(Δ/σ)_max_ = 0.001
112 parameters	Δρ_max_ = 0.78 e Å^−^^3^
3 restraints	Δρ_min_ = −0.25 e Å^−^^3^

### Fractional atomic coordinates and isotropic or equivalent isotropic displacement parameters (Å^2^)

**Table:**

	*x*	*y*	*z*	*U*_iso_*/*U*_eq_	
S1	0.31946 (5)	0.62757 (3)	0.66986 (4)	0.02663 (17)	
O1	0.14241 (15)	0.57550 (9)	0.57815 (14)	0.0388 (3)	
O2	0.44282 (17)	0.61906 (10)	0.59274 (16)	0.0405 (3)	
O3	0.29844 (16)	0.73891 (8)	0.71461 (14)	0.0358 (3)	
O4	0.4472 (2)	0.39499 (10)	0.71737 (15)	0.0439 (3)	
H4	0.523 (2)	0.3448 (13)	0.742 (3)	0.047 (6)*	
N1	0.1913 (2)	0.41948 (15)	0.3680 (2)	0.0466 (4)	
H1	0.132 (3)	0.445 (2)	0.422 (3)	0.076 (8)*	
H2	0.3121 (16)	0.432 (2)	0.410 (3)	0.085 (9)*	
C1	0.4220 (2)	0.55462 (11)	0.85428 (17)	0.0274 (3)	
C2	0.4752 (2)	0.44621 (12)	0.85763 (18)	0.0305 (3)	
C3	0.5534 (2)	0.39234 (12)	1.00446 (19)	0.0308 (3)	
H3	0.5897	0.3200	1.0083	0.037*	
C4	0.1193 (3)	0.4894 (2)	0.2257 (3)	0.0727 (7)	
H4A	0.1489	0.5640	0.2578	0.109*	
H4B	−0.0122	0.4812	0.1710	0.109*	
H4C	0.1741	0.4689	0.1551	0.109*	
C5	0.1563 (3)	0.3028 (2)	0.3375 (4)	0.0769 (8)	
H5A	0.2122	0.2637	0.4372	0.115*	
H5B	0.2081	0.2784	0.2665	0.115*	
H5C	0.0257	0.2898	0.2890	0.115*	

### Atomic displacement parameters (Å^2^)

**Table:**

	*U*^11^	*U*^22^	*U*^33^	*U*^12^	*U*^13^	*U*^23^
S1	0.0296 (2)	0.0240 (2)	0.0307 (3)	−0.00098 (12)	0.01698 (18)	0.00029 (12)
O1	0.0337 (6)	0.0388 (6)	0.0430 (7)	−0.0054 (5)	0.0156 (5)	−0.0072 (5)
O2	0.0418 (7)	0.0465 (7)	0.0445 (7)	0.0048 (5)	0.0293 (6)	0.0084 (5)
O3	0.0463 (6)	0.0230 (5)	0.0402 (6)	0.0005 (4)	0.0205 (5)	0.0012 (4)
O4	0.0667 (9)	0.0330 (6)	0.0303 (6)	0.0166 (6)	0.0192 (6)	−0.0040 (5)
N1	0.0400 (8)	0.0577 (10)	0.0451 (9)	0.0031 (7)	0.0211 (7)	−0.0086 (7)
C1	0.0334 (7)	0.0236 (7)	0.0302 (7)	−0.0008 (5)	0.0183 (6)	0.0001 (5)
C2	0.0409 (8)	0.0251 (7)	0.0300 (8)	0.0011 (6)	0.0194 (6)	−0.0040 (5)
C3	0.0423 (8)	0.0205 (6)	0.0346 (8)	0.0029 (6)	0.0211 (7)	−0.0017 (5)
C4	0.0601 (13)	0.110 (2)	0.0519 (13)	−0.0003 (14)	0.0279 (11)	0.0132 (13)
C5	0.0589 (13)	0.0636 (15)	0.120 (2)	−0.0071 (11)	0.0497 (15)	−0.0276 (14)

### Geometric parameters (Å, °)

**Table:**

S1—O2	1.4462 (12)	C1—C2	1.394 (2)
S1—O1	1.4531 (11)	C2—C3	1.391 (2)
S1—O3	1.4577 (11)	C3—C1^i^	1.391 (2)
S1—C1	1.7799 (15)	C3—H3	0.9300
O4—C2	1.3657 (18)	C4—H4A	0.9600
O4—H4	0.827 (9)	C4—H4B	0.9600
N1—C5	1.463 (3)	C4—H4C	0.9600
N1—C4	1.462 (3)	C5—H5A	0.9600
N1—H1	0.879 (10)	C5—H5B	0.9600
N1—H2	0.890 (10)	C5—H5C	0.9600
C1—C3^i^	1.391 (2)		
			
O2—S1—O1	112.67 (8)	O4—C2—C1	119.56 (14)
O2—S1—O3	113.09 (7)	C3—C2—C1	118.83 (13)
O1—S1—O3	112.00 (7)	C2—C3—C1^i^	120.76 (13)
O2—S1—C1	107.34 (7)	C2—C3—H3	119.6
O1—S1—C1	105.76 (7)	C1^i^—C3—H3	119.6
O3—S1—C1	105.31 (7)	N1—C4—H4A	109.5
C2—O4—H4	106.1 (15)	N1—C4—H4B	109.5
C5—N1—C4	115.6 (2)	H4A—C4—H4B	109.5
C5—N1—H1	110.4 (19)	N1—C4—H4C	109.5
C4—N1—H1	101.2 (19)	H4A—C4—H4C	109.5
C5—N1—H2	110 (2)	H4B—C4—H4C	109.5
C4—N1—H2	103.1 (19)	N1—C5—H5A	109.5
H1—N1—H2	116 (3)	N1—C5—H5B	109.5
C3^i^—C1—C2	120.41 (13)	H5A—C5—H5B	109.5
C3^i^—C1—S1	118.67 (11)	N1—C5—H5C	109.5
C2—C1—S1	120.91 (11)	H5A—C5—H5C	109.5
O4—C2—C3	121.61 (13)	H5B—C5—H5C	109.5
			
O2—S1—C1—C3^i^	126.33 (13)	C3^i^—C1—C2—O4	178.89 (14)
O1—S1—C1—C3^i^	−113.16 (13)	S1—C1—C2—O4	−0.5 (2)
O3—S1—C1—C3^i^	5.58 (14)	C3^i^—C1—C2—C3	−0.1 (3)
O2—S1—C1—C2	−54.28 (14)	S1—C1—C2—C3	−179.49 (11)
O1—S1—C1—C2	66.23 (14)	O4—C2—C3—C1^i^	−178.87 (14)
O3—S1—C1—C2	−175.03 (12)	C1—C2—C3—C1^i^	0.1 (3)

Symmetry codes: (i) −*x*+1, −*y*+1, −*z*+2.

### Hydrogen-bond geometry (Å, °)

**Table:**

*D*—H···*A*	*D*—H	H···*A*	*D*···*A*	*D*—H···*A*
O4—H4···O3^ii^	0.83 (1)	1.85 (1)	2.670 (2)	175 (2)
N1—H1···O1	0.88 (1)	2.13 (2)	2.866 (2)	140 (2)
N1—H1···O1^iii^	0.88 (1)	2.21 (2)	2.921 (2)	138 (2)
N1—H2···O2^iv^	0.89 (1)	2.07 (2)	2.837 (2)	143 (3)

Symmetry codes: (ii) −*x*+1, *y*−1/2, −*z*+3/2; (iii) −*x*, −*y*+1, −*z*+1; (iv) −*x*+1, −*y*+1, −*z*+1.

## References

[bb1] Barbour, L. J. (2001). *J. Supramol. Chem.* **1**, 189–191.

[bb2] Higashi, T. (1995). *ABSCOR* Rigaku Corporation, Tokyo, Japan.

[bb3] Rigaku (1998). *RAPID-AUTO* Rigaku Corporation, Tokyo, Japan.

[bb4] Rigaku/MSC (2002). *CrystalClear* Rigaku/MSC Inc., The Woodlands, Texas, USA.

[bb5] Sheldrick, G. M. (2008). *Acta Cryst.* A**64**, 112–122. 10.1107/S0108767307043930 18156677

[bb6] Solans, X., Plana, F. & Font-Altaba, M. (1982). *Acta Cryst.* B**38**, 651–653.

[bb7] Westrip, S. P. (2010). *J. Appl. Cryst.* **43**, 920–925.

